# Fracture Behaviour of Basalt Fibre-Reinforced Lightweight Geopolymer Concrete: A Multidimensional Analysis

**DOI:** 10.3390/ma18153549

**Published:** 2025-07-29

**Authors:** Jutao Tao, Mingxia Jing, Qingshun Yang, Feng Liang

**Affiliations:** 1 Department of Civil Engineering, Qinghai University, Xining 810016, China; 15023466629@163.com (J.T.); yqss112@163.com (Q.Y.); liangf0512@163.com (F.L.); 2 Key Laboratory of Building Energy-Saving Materials and Engineering Safety of Qinghai Province, Xining 810016, China

**Keywords:** basalt fibre, geopolymer concrete, polystyrene particles, XFEM, fracture performance

## Abstract

This study introduced basalt fibres as a reinforcing material and employed notched beam three-point bending tests combined with digital image correlation (DIC) technology to comprehensively evaluate key fracture parameters—namely, initial fracture toughness, unstable fracture toughness, fracture energy, and ductility index—of expanded polystyrene (EPS)-based geopolymer concrete with different mix proportions. The results demonstrate that the optimal fracture performance was achieved when the basalt fibre volume content was 0.4% and the EPS content was 20%, resulting in respective increases of 12.07%, 28.73%, 98.92%, and 111.27% in the above parameters. To investigate the toughening mechanisms, scanning electron microscopy was used to observe the fibre–matrix interfacial bonding and crack morphology, while X-ray micro-computed tomography enabled detailed three-dimensional visualisation of internal porosity and crack development, confirming the crack-bridging and energy-dissipating roles of basalt fibres. Furthermore, the crack propagation process was simulated using the extended finite element method, and the evolution of fracture-related parameters was quantitatively analysed using a linear superposition progressive assumption. A simplified predictive model was proposed to estimate fracture toughness and fracture energy based on the initial cracking load, peak load, and compressive strength. The findings provide theoretical support and practical guidance for the engineering application of basalt fibre-reinforced EPS-based geopolymer lightweight concrete.

## 1. Introduction

Concrete plays an important role in urban construction. However, the manufacturing process of ordinary Portland cement, which acts as the principal binding agent in traditional concrete, is a major contributor to global CO_2_ emissions, accounting for approximately 6–7% of total worldwide CO_2_ emissions [1,2,3]. Geopolymer concrete, which replaces conventional cementitious materials with geopolymer binders, is a promising solution for mitigating the carbon footprint associated with concrete production.

Moradikhou and Kim et al. [4,5] developed ambient-cured geopolymer concrete with slag as a supplementary cementitious material to address the challenge of heat curing. However, it is difficult to implement on-site. Provis et al. [6] investigated fly ash-based geopolymer concrete and optimised the fly ash–alkali activator ratio, determining that a ratio between 0.5 and 0.7 significantly enhanced the strength of the fly ash-based geopolymer concrete.

Kanagaraj et al. [7] investigated the setting time of geopolymer concrete, which is a crucial factor for ensuring adequate workability and proper curing during construction. They highlighted that the binder composition, particularly low-Ca fly ash and mineral admixtures, strongly influences the setting characteristics. Aguila et al. [8] conducted experimental studies on low-density geopolymer concrete, incorporating 100% and 75% high-reactivity mineral admixtures, with fly ash proportions of 0% and 25% as binders, demonstrating superior thermal insulation properties. Sanjayan et al. [9] examined lightweight geopolymer concrete derived from fly ash, emphasising the significance of alkali activator composition, particularly the ratio of Na_2_SiO_3_ to NaOH, and the relationship between the alkaline solution and the binder in optimising material performance.

Due to its low density, hydrophobicity, thermal insulation properties, non-absorbent nature, and cost-effectiveness, expanded polystyrene (EPS) can be used partially or wholly as a replacement for natural aggregates in concrete to produce lightweight concrete materials, thereby reducing its environmental impact [10]. Consequently, incorporating different amounts of modified or unmodified EPS particles into geopolymer concrete enables the production of low-density concrete with varying densities. EPS-based geopolymer concrete with adequate strength and durability can be applied in traditional building construction, subgrade materials, and floating and offshore structures, as well as energy-absorbing structures subjected to impact loads [11]. However, Shirai et al. [12] showed that geopolymer concrete is prone to early-age cracking and brittle failure, significantly reducing the mechanical performance. Bhutta et al. [13] proposed that incorporating fibres could substantially enhance the compressive strength, splitting tensile strength, and flexural toughness of geopolymer concrete. Basalt fibres have several applications in engineering fields owing to their chemical stability, superior compatibility with concrete, cost-effectiveness, and environmentally sustainable attributes [14,15,16].

Pham et al. [17] assessed the feasibility of basalt fibre-reinforced geopolymer concrete as a potential substitute for conventional concrete. Their findings revealed that basalt fibres enhanced the fibre–matrix transition zone, and an appropriate increase in fibre content contributed to enhanced setting times, compressive strength, and flexural strength in geopolymer concrete. Şahin et al. [18] explored the impact of basalt fibres on geopolymer matrices, reporting that their inclusion boosted the compressive strength and flexural strength by approximately 25% and 50%, respectively. Experimental studies have consistently demonstrated that basalt fibre-reinforced geopolymer concrete exhibited outstanding mechanical properties [18,19,20,21,22,23,24]. Despite these advantages, research focusing on the fracture behaviour of this concrete remains relatively scarce. Therefore, a more thorough investigation of the fracture characteristics of EPS-based lightweight geopolymer concrete incorporating basalt fibres is essential. Fracture toughness and fracture energy serve as critical parameters for evaluating the fracture resistance of concrete, but real-time crack propagation monitoring is equally important. Digital image correlation (DIC) techniques have been used to analyse the strain and displacement fields in EPS-based lightweight geopolymer concrete [23,24,25,26].

To comprehensively investigate the fracture behaviour of basalt fibre-reinforced EPS-based lightweight geopolymer concrete, this study integrated experiments and numerical simulations to systematically analyse the crack propagation mechanism and fracture toughness. A notched beam three-point bending test was performed to evaluate the fracture toughness of the material. Scanning electron microscopy (SEM) was employed to examine the fracture surface morphology, providing insights into the interfacial bonding characteristics between the basalt fibres and the geopolymer matrix, as well as the failure mechanisms. Furthermore, X-ray computed tomography (CT) was utilised to construct a three-dimensional (3D) representation of the internal crack morphology, facilitating a quantitative analysis of crack network formation and damage accumulation. Building on the experimental findings, this study implemented the extended finite element method (XFEM) to establish a numerical model for simulating crack propagation behaviour. Subsequently, the numerical outcomes were compared with the experimental data to assess the model’s accuracy and investigate the fracture mechanisms and key influencing factors of basalt fibre-reinforced EPS-based geopolymer concrete. By comparing the experimental results with those of numerical simulations, this approach validates the reliability of the experimental results and examines the relationships among compressive strength, peak load, and crack initiation load through curve-fitting techniques. This analysis helps elucidate the underlying fracture mechanisms, ultimately enabling the assessment of fracture performance based on concrete compressive strength.

To assess the toughening effect of basalt fibres in EPS-based lightweight geopolymer concretes, this study examined the fracture performance at fibre volume fractions of 0.4% and 0.6% [27]. By integrating three-point bending tests with DIC technology, key fracture parameters throughout the entire process of crack initiation, propagation, and instability were accurately captured. SEM and micro-CT were employed to conduct detailed analyses of the microstructural features and crack evolution behaviour, revealing the crack propagation modes and damage evolution mechanisms induced by fibre reinforcement. Additionally, XFEM was utilised to simulate crack development numerically, further enhancing the understanding of fracture behaviour under different mix proportions. Based on the experimental results, a simplified predictive model was established by correlating the initial cracking load, peak load, and compressive strength, allowing for efficient estimation of fracture toughness and fracture energy. This study not only identifies the optimal combination of basalt fibre and EPS particle contents but also provides a theoretical foundation and technical reference for the practical application of this lightweight geopolymer concrete in green buildings, thermal insulation walls, and lightweight structural components.

## 2. Overview of the Three-Point Bending Test

### 2.1. Specimen Design

Grade I fly ash was used in this study. The ground granulated blast-furnace slag conformed to the GB/T203 [28] standard and was classified as an S95-grade slag powder, with a moisture content of 0.43% and a density of 3.1 g/cm^3^. The EPS particles had a size range of 2–5 mm, an average diameter of 4.1 mm, and a density of 18.3 kg/m^3^. To enhance the mechanical properties, 9 mm long basalt fibres were incorporated as reinforcement. The alkaline activator consisted of NaOH pellets (Xilong Scientific, Shantou, China; purity = 98%) and a Na_2_SiO_3_ solution (Shanghai Zhecai IoT Co., Shanghai, China). The NaOH pellets were of guaranteed reagent analytical grade and complied with the GB/T 629-1997 [29] standard. The key parameters of Na_2_SiO_3_ are listed in Table 1. Natural river sand was selected as the fine aggregate, which exhibited a uniform particle size distribution and was classified as well-graded sand. According to ASTM C128-15 [30], the sand was oven-dried at 105 °C for 48 h prior to testing to ensure a saturated surface dry (SSD) condition. Additionally, to improve the performance of the EPS-based geopolymer concrete, a polycarboxylate-based high-performance water reducer was introduced at a dosage of 1.5% by weight of the binder materials [31].

In this experiment, EPS and basalt fibre volume fractions were used as variables. The mix proportion designs of the test specimens are listed in Table 2. For example, the notation LEGC20BF04 represents an EPS content of 20% and a basalt fibre content of 0.4%.

**Figure 1:**
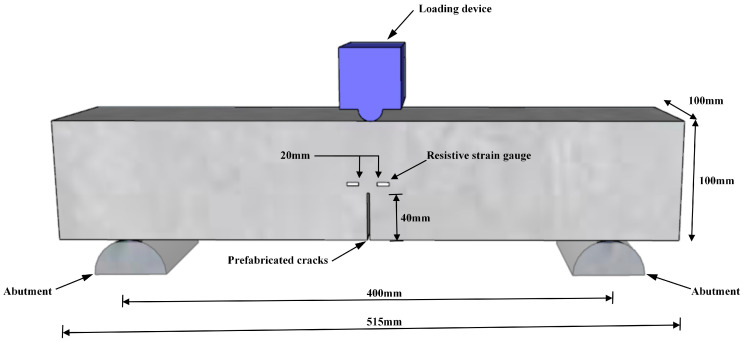
Engineering drawing of specimen.

Twenty-seven specimens were fabricated for the experiment and divided into three groups. Each specimen measured 100 mm × 100 mm × 515 mm, maintaining a span-to-height ratio of 4. The initial notch length (a_0_) was set to 40 mm. The detailed specimen design and dimensions are illustrated in Figure 1.

### 2.2. Specimen Preparation Process

**Figure 2:**
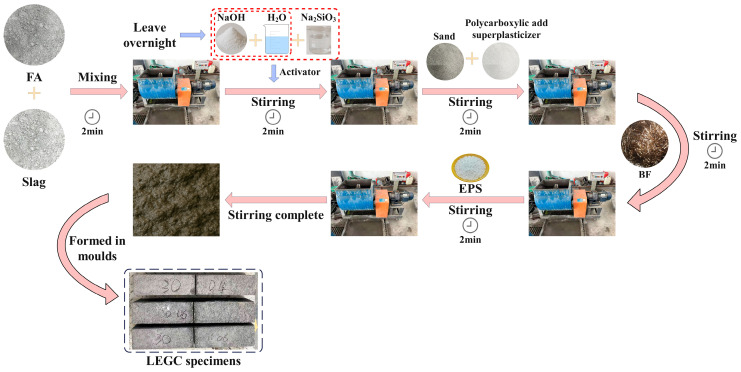
Flow chart of specimen preparation.

The preparation sequence of the lightweight expanded polystyrene geopolymer concrete (LEGC) under ambient curing conditions was as follows: (1) Dry mixing: Fly ash (FA) and ground granulated blast furnace slag (slag) were dry-mixed in a cement mortar mixer for 2 min; (2) Alkaline activator addition: After 2 min, a pre-mixed solution of NaOH (left for one day) and Na_2_SiO_3_ was added to the mixer and stirred for another 2 min; (3) Sand and superplasticiser addition: After a total mixing of 4 min, sand and a polycarboxylate-based high-performance water reducer were added and mixed for 2 min; (4) Basalt fibre addition: Basalt fibres were introduced and mixed for 2 min; (5) EPS particle addition: EPS particles were added and mixed for 2 min. The total mixing process lasted for 10 min. The LEGC preparation process is illustrated in Figure 2.

Before casting the concrete, a 2 mm thick steel plate coated with an oil-based release agent on both sides was secured inside the mould. After an initial setting time of 3 h, the steel plate was unfastened, and upon de-moulding, it was carefully removed to create a preformed notch. Once the specimens were cast, they were stored at 20 ± 5 °C for 3 days. After de-moulding, the specimens were placed in a standard curing environment and maintained under controlled conditions for 28 days.

## 3. Experimental Results and Analysis

### 3.1. Fracture Process Analysis

**Figure 3:**
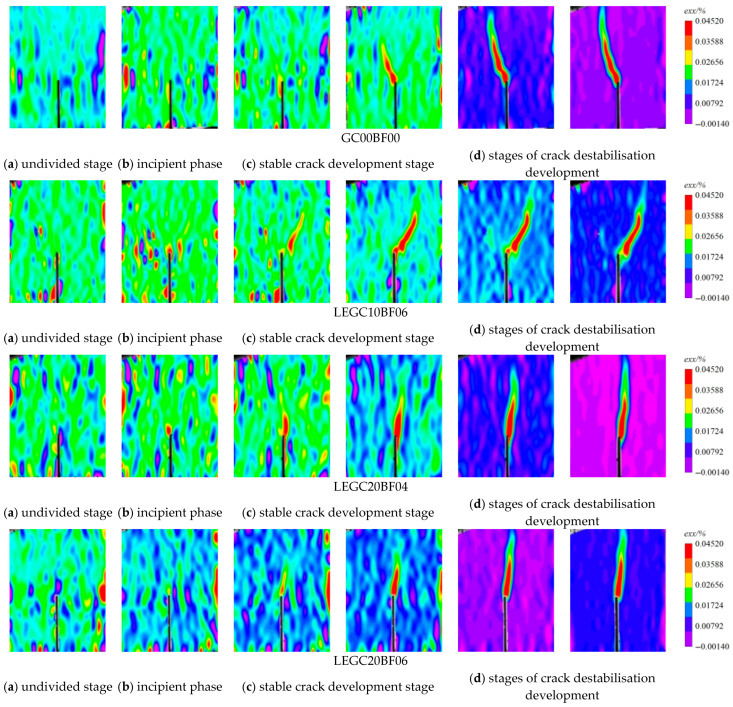
Crack propagation in fracture process zone of specimen.

Figure 3 illustrates the strain field distribution and failure patterns of the LEGC specimens with different mix proportions. Prior to crack initiation, the entire concrete specimen sustained the applied load, with the strain field predominantly appearing green. As the crack began to propagate, a localised strain concentration zone became evident at the crack tip. The development of the strain concentration zone in the LEGC containing basalt fibres and EPS particles was more irregular than that in the fibre- and EPS-free GC. The irregular distribution of basalt fibres and EPS particles contributed to the unpredictability of the crack propagation path, whereas the effectiveness of basalt fibres in impeding macrocrack expansion is particularly pronounced.

The development pattern of the FPZ at the free crack front in all specimens corresponded to the progressive stages of the P-CMOD curve. During propagation, the crack was continuously obstructed by the geopolymer matrix, sand, and EPS particles, causing it to extend tortuously along the direction of the principal stress, ultimately forming a single narrow main crack. The FPZ shape of specimen GC is generally a narrow “I” shape, tapering into a triangular form at the crack tip. Before reaching the peak load, the crack remained relatively small. Once the peak load was attained, it propagated rapidly, resulting in a significantly reduced load-bearing capacity and distinct brittle behaviour. In contrast, the LEGC specimens exhibited higher strain levels after loading than the GC specimens. The bridging effect of the basalt fibres, coupled with the energy absorption properties of the EPS particles, enabled the geopolymer matrix in the LEGC to continue transmitting partial stress even after the initial cracking and peak load phases. This resulted in strain softening rather than immediate catastrophic failure. Furthermore, no completely penetrating cracks were detected at the instability point. Overall, the incorporation of basalt fibres and EPS particles significantly enhanced the toughness of the geopolymer concrete, effectively reducing its brittleness.

### 3.2. Load Deflection Curve

A clip-on extensometer was utilised to capture the P-CMOD and P–δ curves. All nine specimen groups exhibited comparable trends following a three-stage fracture process, which included linear elastic, stable crack propagation, and unstable crack propagation phases.

**Figure 4:**
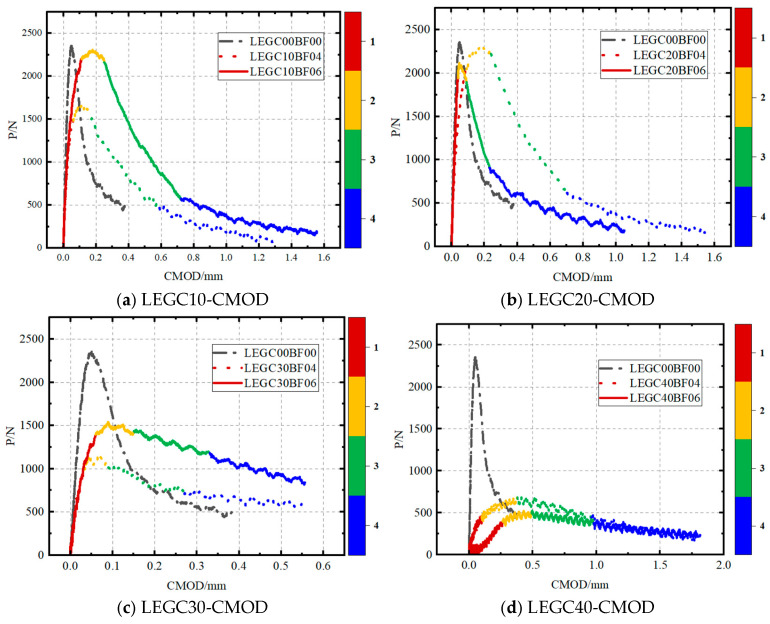
Load–crack mouth opening displacement (P-CMOD) curves of concretes.

As shown in Figure 4, the P-CMOD curve can be segmented into four distinct stages: (1) the load increases linearly with a marginal increase in crack mouth opening displacement (CMOD); (2) as the load nears its peak value, the pre-existing crack starts propagating; (3) during the unloading phase, the CMOD expands rapidly; and (4) the CMOD continues to increase steadily, while the load exhibits a minor increment. When comparing the GC00BF00 group (without basalt fibres) to the LEGC specimens, both fibre-reinforced and non-fibre-reinforced variants exhibit comparable behaviour in the linear phase. This similarity emerged because, at this stage, the specimen did not undergo significant damage, and the relative positioning between the fibres and the concrete matrix remains unchanged. Consequently, no frictional resistance was generated, and the load was entirely carried by the concrete matrix [32]. Once crack propagation began, the basalt fibres slipped relative to the concrete matrix and acted as a mechanical interlock [33], bridging the cracks and enhancing the fracture resistance. Additionally, owing to the cyclic replacement of the failed fibres with new load-bearing fibres, the load exhibited a marginal increase in the final stage as the CMOD continued to increase, contributing to improved ductility. As the basalt fibre volume fraction increased, the enhancement in ductility became more pronounced.

Basalt fibres primarily influenced the second and third stages of the P-CMOD curve. Compared to the GC without fibres, the LEGC containing basalt fibres and EPS particles exhibited a slower load growth rate in the second stage. Additionally, during the unloading stage, the LEGC with basalt fibres and EPS particles showed a more gradual failure mode because when fibres in the failure zone were pulled out or broken, a fibre network formed by intact fibres at the crack tip provided strong resistance to crack propagation. Consequently, the ductility of LEGC was significantly improved. As shown in Table 3, at the onset of cracking, the LEGC specimens incorporating basalt fibres exhibit significantly higher crack mouth opening displacement values compared to the GC00BF00 specimens. This indicates that the LEGC specimens did not fail immediately after crack initiation, effectively delaying brittle fracture and enhancing post-cracking stability. Moreover, the EPS particles in the LEGC mixture exhibit excellent shock-absorbing and energy-dissipating capabilities. This is evidenced by the area enclosed by the P–δ curves, where all LEGC specimens with basalt fibre additions show larger areas than the GC00BF00 specimens. Notably, the energy absorption capacity of LEGC30BF06 is approximately 2.5 times that of GC00BF00. As the EPS particles participate in the geopolymerisation process and form hydration products with the matrix, they become an integral part of the concrete microstructure, thereby enhancing the overall ductility of the material.

**Figure 5:**
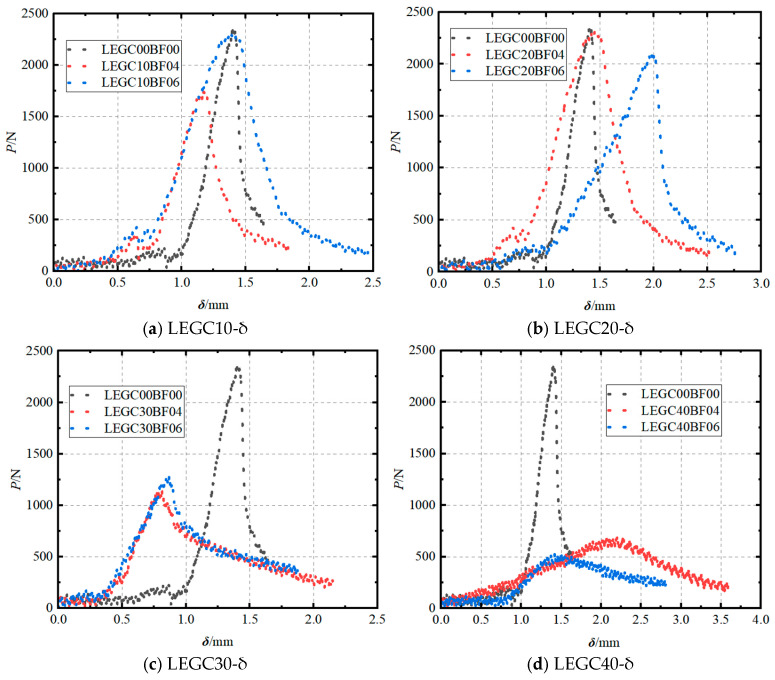
Load–deflection (P–δ) curves of concretes.

The P–δ curves of LEGC with different mix proportions (Figure 5) exhibited a stepped variation trend, indicating the non-uniform cracking process in LEGC. During fracture propagation, when the first fibre bridged the crack, it temporarily halted crack growth. At this stage, the load remained constant despite the increase in displacement until the bridging effect of the fibre failed. Consequently, LEGC containing basalt fibres and EPS particles showed a longer relative stabilisation phase in the P–δ curve. This process significantly enhances the toughness of LEGC, requiring the material to store more fracture energy as the crack gradually propagates upward. This trend correlates well with the behaviour observed in the P-CMOD curve.

### 3.3. Determination of Crack Initiation Load

**Figure 6:**
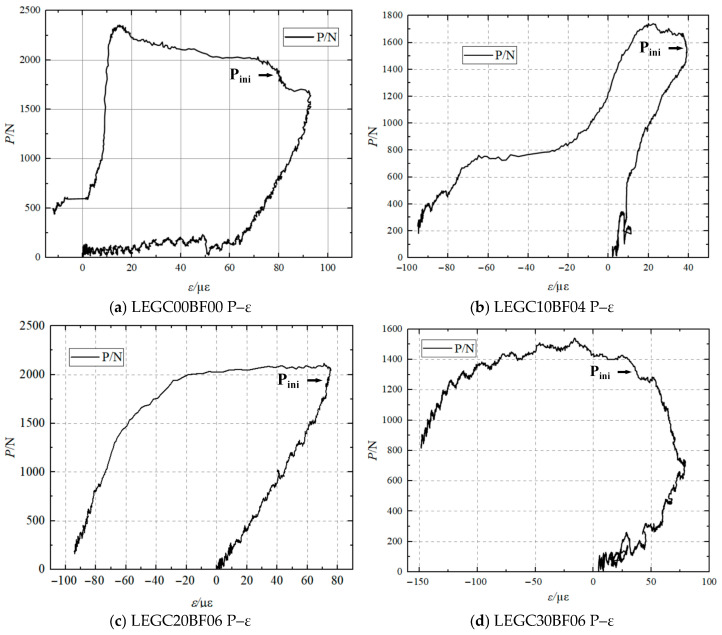
Load–strain (P–*ε*) curves of concretes.

Figure 6 presents the load–strain (P–ε) curves of LEGC specimens with different mix proportions obtained from strain gauges. Once the crack tip was initiated, the concrete adjacent to the crack exhibited a distinct strain hysteresis effect resulting from the strain release. This phenomenon enabled the accurate and intuitive determination of the initial cracking load (P_ini_), thereby offering essential data support for the fracture characterisation of LEGC. The specific values of the initial cracking load are presented in Table 3.

## 4. Microanalysis of Fibre-Reinforced Fracture Properties

### 4.1. X-CT-Based Structural Analysis of Concrete Cracks and Voids

X-ray micro-computed tomography (X-CT) is highly effective in visualising the internal structure and phase distribution of cement-based materials, including concrete. For example, the SkyScan1173 tomography system has been successfully utilised to examine the fracture evolution of concrete under various mechanical testing conditions, including the three-point bending of ordinary concrete [31], tensile splitting [32], uniaxial compression [33], and compressive fatigue [34]. Additionally, it has been employed for continuous scanning during three-point bending tests [35] and investigating the wedge-splitting fracture behaviour in steel fibre- and basalt fibre-reinforced concrete [36,37]. Thus, X-CT has become a widely adopted fracture research technique among other researchers [38,39,40,41,42,43,44,45].

Given the substantial dimensions of the concrete beam used in the experiment, the central core region was selected and cut into cubic samples with final dimensions of 100 mm × 100 mm × 100 mm, which contained EPS particles with diameters ranging from 2 to 5 mm. The concrete cube sample was scanned using the NanoVoxel-0 series X-ray 3D scanning system, which included an X-ray source, a rotating control platform, and a scanner. The system was operated at a magnification of 150×, achieving an image resolution of 45.775 μm. For basalt fibre-reinforced EPS geopolymer lightweight concrete, the cross-section was horizontally scanned layer-wise from top to bottom, resulting in 1441 consecutive two-dimensional (2D) slice images.

**Figure 7:**
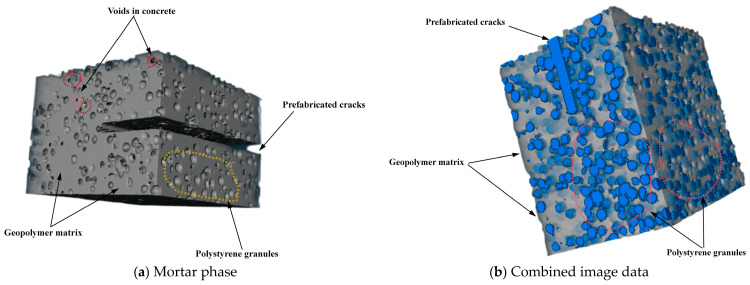
Concrete matrix and EPS particles under CT scanning.

During scanning, the instrument operated at a voltage of 120 kV and a current of 145 mA, with an image exposure time of 0.35 s. The X-CT images were processed using Avizo9.0 visualisation software, while enabled the accurate reconstruction of the concrete model with enhanced structural detail. By applying a watershed segmentation algorithm, we determined the optimal segmentation thresholds for voids and the geopolymer matrix in the basalt fibre-reinforced EPS geopolymer concrete samples to be 27,942 and 32,886, respectively. Figure 7 displays a representative X-CT scan slice along with the threshold segmentation effect of the concrete sample, where the blue regions represent the EPS granules and the grey region represents the geopolymer matrix.

**Figure 8:**
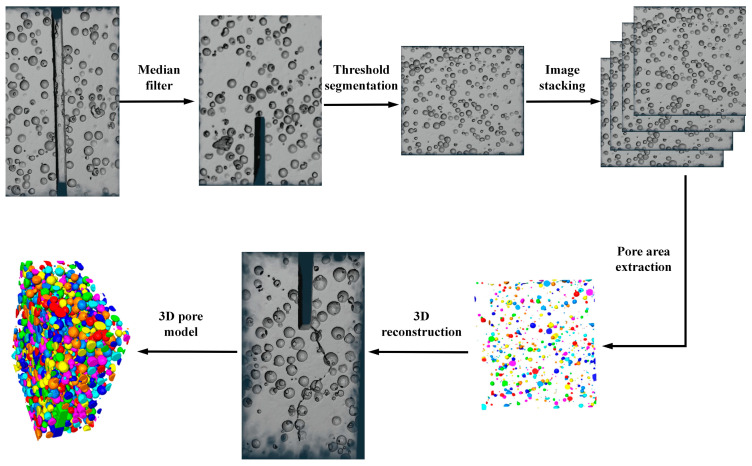
Acquisition and segmentation of X-ray micro-computed tomography (CT) image datasets and model reconstruction.

To enhance the clarity of the 3D pore structure and EPS distribution in the lightweight concrete, a median filtering algorithm was applied to the X-CT slices for noise reduction. Using the processed images, the threshold segmentation technique (Figure 8) was employed to precisely differentiate the voids, EPS particles, and geopolymer matrix.

By stacking the images, the pore regions were successfully isolated, enabling the reconstruction of the 3D model. This model was used to construct a 3D pore structure model, which served as the basis for the quantitative analysis of the pore characteristics. Meanwhile, the reconstructed model revealed critical features, such as the size and distribution of voids and EPS particles. It provided essential information for evaluating the properties of this type of concrete.

The EPS particles were uniformly distributed with varying diameters, forming an intricate pattern. The small spheres of different colours in Figure 7 represent EPS particles of different sizes. Similarly, in the reconstructed 3D pore model, the differently coloured spheres represent voids with varying diameters. The consistency of the void structure is a key factor influencing the mechanical properties of EPS geopolymer lightweight concrete. Concrete with higher porosity and non-uniform void distribution tends to have lower mechanical strength. As shown in Figure 8, the voids in this concrete are relatively uniformly distributed, which contributed significantly to the improvement in its fracture performance. Additionally, the uniform distribution of EPS particles enabled the concrete to absorb more energy, resulting in greater ductility during fracture.

**Figure 9:**
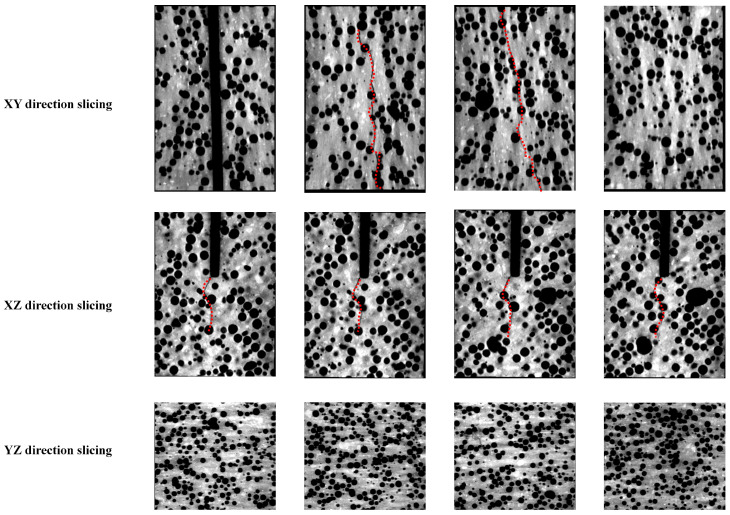
Sections in all directions under CT scanning. Where the red dotted line indicates the path of development of the cracks.

Crack propagation in concrete is a key factor affecting its fracture performance. The development of cracks influences both the fracture energy and ductility index, which are essential parameters for characterising the fracture behaviour of concrete. To analyse crack propagation trends in the basalt fibre-reinforced EPS geopolymer lightweight concrete, 2D CT slices of the concrete samples were examined on the XY, XZ, and YZ planes. As shown in Figure 9, the EPS particles in the concrete sample are uniformly distributed. This distribution altered the original crack propagation path, significantly enhancing the fracture performance of the concrete. Observations of the YZ plane indicated that EPS particles were uniformly distributed across each section from left to right. By analysing the XY and XZ planes, the cracks evidently did not penetrate the entire height of the concrete cross-section; instead, they stopped at a certain height when the concrete reached failure. The XZ plane further revealed that crack propagation in the lightweight concrete did not follow a simple I-shaped pattern. Instead, cracks developed along different paths on each plane. This phenomenon is mainly attributed to the influence of EPS particles on the crack paths, along with the strong bridging action of the basalt fibres within the geopolymer matrix. Consequently, crack propagation does not occur in one direction, which corroborates the experimental findings.

### 4.2. SEM Scanning Analysis

**Figure 10:**
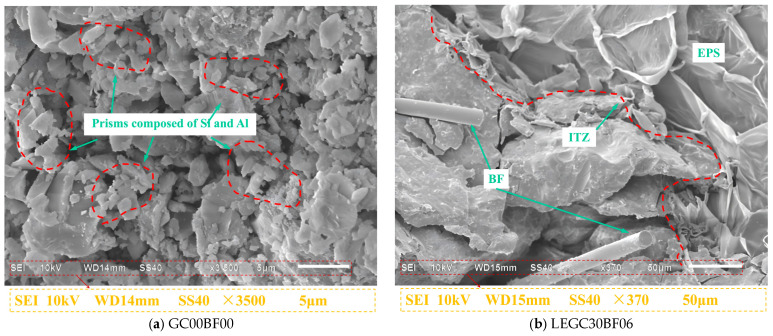
SEM images of specimens GC00BF00 and LEGC30BF06 at day 28.

SEM images of the hydration products of the 28-day cured GC00BF00 and LEGC30BF06 specimens are shown in Figure 10. At a magnification of 3500×, the micrographs reveal the formation of prismatic structures due to the reaction of the fly ash particles. These prisms were primarily composed of Si and Al, suggesting that the formation of this binder was predominantly governed by Si-O and Al-O bonds. This finding is consistent with the conclusions of Siddique and Mehta [46]. The SEM image of LEGC30BF06 shows that the basalt fibres exhibited no significant deformation and that the geopolymer matrix remained intact. Furthermore, the geopolymer matrix exhibited a strong bond with the EPS particles, contributing to the enhanced durability and long-term performance of the concrete. These findings confirm that the robust interplay among basalt fibres, EPS particles, and the geopolymer matrix plays a crucial role in enhancing the fracture resistance and ductility of concrete.

**Figure 11:**
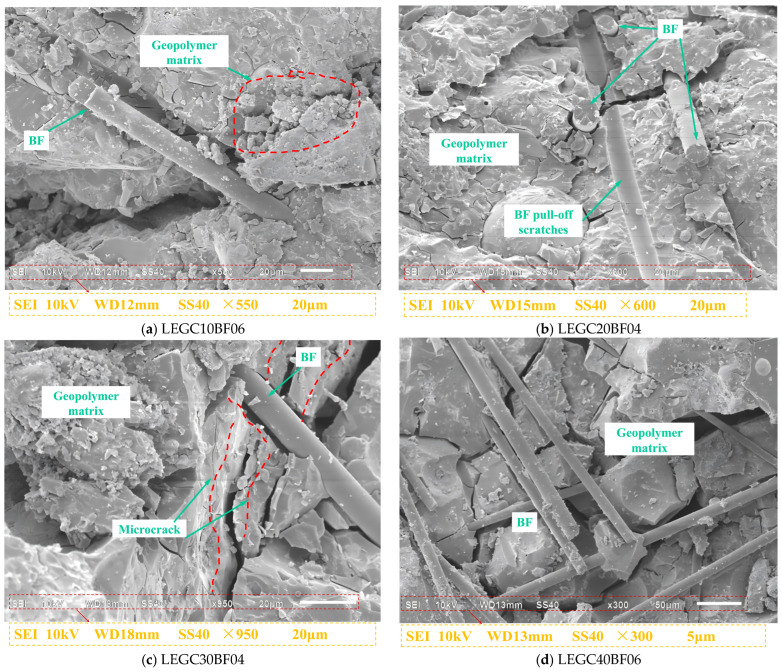
SEM images of microstructure and morphology of ITZ in concretes.

The distribution of basalt fibres in LEGC is shown in Figure 11. During crack propagation, the cracks were blocked by basalt fibres. When the bond stress between the basalt fibres and the concrete matrix was sufficiently high, the available energy was inadequate to induce fibre pullout or rupture. Consequently, basalt fibres inhibited crack propagation through their bridging effects. When the bond stress was insufficient, the fibres lost adhesion and were pulled out, with some mortar adhering to their surface. The deformation or fracture of the basalt fibres absorbed a substantial amount of energy. When the fibres were weaker, they underwent deformation or breakage under an applied energy, forming voids and gaps between the fibres and the concrete matrix. Thus, their bonding strength was weakened. At this stage, the bridging effect, along with fibre pullout and rupture, reduced the crack length and width, ultimately enhancing the bonding quality of the concrete matrix and improving the peak load capacity, fracture toughness, and fracture energy. Bridging and fibre rupture were observed in all the basalt fibre-reinforced EPS geopolymer lightweight concrete specimens. However, fibre clustering was predominantly detected in specimens with a higher fibre content (0.6%). The formation of fibre clusters weakened the bond stress, thereby diminishing the reinforcement effectiveness of the basalt fibres.

SEM was utilised to investigate the fibre–matrix interfacial characteristics and crack propagation paths, aiming to elucidate the fundamental mechanism by which fibre bridging enhances the macroscopic fracture energy. Although mechanical testing and numerical simulations have demonstrated a notable improvement in fracture performance with fibre incorporation, the underlying mechanism is further clarified at the microscale. SEM analysis reveals that the fibres form a spatially interwoven three-dimensional network within the matrix. As cracks propagate into fibre-dense regions, the interfacial bonding induces a bridging effect, compelling crack deflection or diversion around the fibres, thereby enhancing energy dissipation. Distinct evidence of fibre pullout traces and interfacial debonding confirms that the fibres undergo a multi-stage energy absorption process—from elastic load-bearing to plastic sliding—during fracture progression. These microscale behaviours are directly correlated with the experimentally observed increases in macroscopic fracture energy, further validating the consistency of experimental data and numerical predictions. Based on these results, a cross-scale reinforcement mechanism is proposed, which involves fibre bridging, crack deflection, and energy dissipation. This mechanism provides valuable microstructural insights that can inform the optimisation of key parameters, including fibre content and interfacial modification methods.

## 5. Fracture Toughness

Fracture toughness characterises the resistance of a material to stress intensity and is determined using the fracture toughness formula outlined in the double-K fracture criterion for concrete crack propagation, as proposed by Xu [47]. According to the P–δ curve, the three-point bending method recommended by the International Union of Laboratories and Experts in Construction Materials, Systems, and Structures (RILEM) [48] was employed to calculate the fracture energy (G_F_).

**Figure 12:**
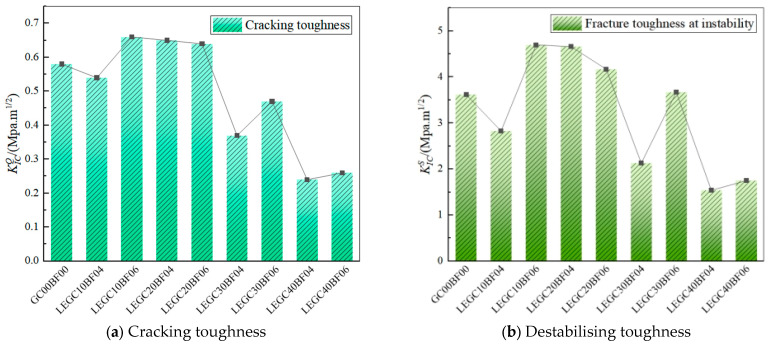
Cracking toughness and destabilising toughness of specimens.

Utilising the parameters derived from the P-CMOD and P–δ curves, the double-K fracture parameters and fracture energy for the nine specimen groups for 28 days were determined. The initial and unstable fracture toughness values are illustrated in Figure 12. As indicated in the figure, when compared to that of GC00BF00, the unstable fracture toughness of LEGC10BF06, LEGC20BF04, LEGC20BF06, and LEGC30BF06 increased by 29.83%, 28.73%, 15.19%, and 1.38%, respectively. Similarly, the initial fracture toughness of LEGC10BF06, LEGC20BF04, and LEGC20BF06 improved by 13.91%, 12.07%, and 10.34%, respectively. However, for the other specimens, both the initial and unstable fracture toughness declined, especially for LEGC40BF04 and LEGC40BF06, where the EPS content reached 40%. This behaviour is primarily attributed to the mechanical characteristics of EPS particles, which exhibit significantly lower strength and stiffness than natural aggregates.

When fine aggregates are replaced with EPS, the original sand–gravel framework is weakened, thereby reducing the load-bearing capacity of the material. Although EPS particles exhibited excellent plasticity and toughness and provided energy absorption and damping effects, they had a relatively weak ITZ with the geopolymer matrix. Consequently, under tensile stress, EPS particles detached from the matrix before rupturing, offering limited enhancement of the fracture toughness. Upon reaching the peak load, the geopolymer matrix began to fail. Randomly distributed basalt fibres, with their unique surface morphology, formed a network structure that effectively prevented EPS particle slippage, thereby delaying macrocrack propagation and significantly improving both the unstable and initial fracture toughness. However, with a further increase in the EPS content, both the unstable and initial fracture toughness exhibited a declining trend.

Although the inclusion of basalt fibres enhanced the fracture resistance, the weakening effect of EPS particles outweighed the bridging effect of the former. This phenomenon is mainly attributed to the substantially lower strength of EPS particles compared with that of natural aggregates, which resulted in the development of additional weak zones within the specimen, ultimately diminishing the overall tensile strength.

**Figure 13:**
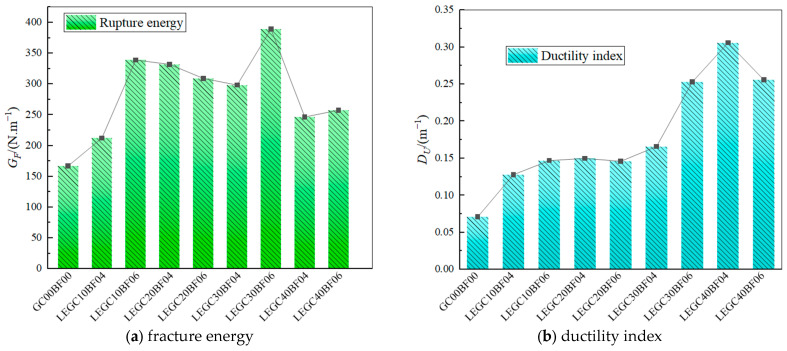
Fracture energies and ductility indices of specimens.

The fracture energies and ductility indices of the specimens are presented in Figure 13. Compared to the trend in GC00BF00, the incorporation of basalt fibres and EPS particles significantly enhanced both the fracture energy and ductility index of all specimens, from LEGC10BF04 to LEGC40BF06. For instance, the ductility indices of LEGC20BF04 and LEGC30BF06 increased by 111.27% and 256.34%, respectively, while their fracture energy increased by 98.92% and 133.18%, respectively. During crack propagation, the brittle nature of GC00BF00 caused it to fracture rapidly, whereas the toughness of the EPS particles allowed them to absorb energy, thereby hindering further crack propagation.

In addition, basalt fibres exhibited a strong crack-arresting effect. As cracks propagated within the concrete matrix, the bridging effect of these fibres generated tensile stress, facilitating additional energy absorption and hindering further crack expansion. Although the load-bearing capacities of LEGC30 and LEGC40 were relatively low, upon reaching their peak load, the crack-arresting function of EPS particles and basalt fibres became evident. This prolonged the transition from instability to failure, notably enhancing both the fracture energy and ductility index. The synergistic effect of EPS particles and basalt fibres substantially enhanced the concrete ductility and energy dissipation capacity, effectively mitigating the brittle behaviour of the material.

In summary, replacing natural aggregates with EPS particles led to varying degrees of reduction in the compressive strength, initial fracture toughness, and peak load of concrete. However, when the EPS and fibre volume fractions were 20% and 0.4%, respectively, LEGC20BF04 exhibited superior fracture performance compared to GC00BF00, with more balanced and stable fracture properties than the other specimens. This is attributed to the synergistic effect of EPS particles and basalt fibres, which enhanced the deformation capacity and load-bearing ability of concrete. At a low EPS content, the optimal basalt fibre dosage was 0.4%. When the fibre content surpassed 0.4%, the fracture performance declined because of poor fibre dispersion within the specimen, causing fibre agglomeration and creating weak zones. Conversely, at a higher EPS content, the optimal basalt fibre dosage increased to 0.6%. In low-EPS concrete, basalt fibres are mostly linearly distributed, leading to a simplified crack propagation path. Surpassing the optimal fibre content does not modify the linear crack trajectory; instead, it leads to the formation of additional weak zones due to excessive fibre aggregation. As the EPS content increases, the randomly dispersed EPS particles force the fibres into a wavy distribution, complicating the crack propagation path. This makes fibre pullout more difficult, resulting in a shift in the optimal basalt fibre dosage to a higher EPS content.

## 6. Extended Finite Element Analysis

In the finite element simulation of concrete structures, it is essential to account for the complexity of failure mechanisms and crack propagation patterns. Given the high material variability and heterogeneity at the mesoscale in EPS-based geopolymer lightweight concrete, conventional constitutive models may not adequately capture its discrete fracture behaviour. To address this, the present study employed the extended finite element method (XFEM), which enables more accurate modelling of discontinuities and fracture evolution without requiring mesh refinement along crack paths.

### 6.1. Finite Element Model

**Figure 14:**
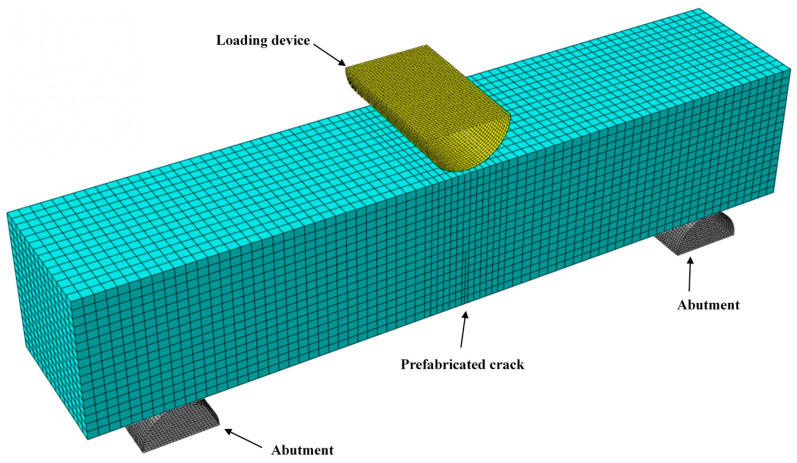
ABAQUS numerical model.

A bending beam simulation was performed using the XFEM on ABAQUS6.14. The model dimensions were consistent with those of the experimental setup. The model parameters are listed in Table 4. Simply supported constraints were applied at both support positions on the lower surface, 200 mm from the mid-span. The simulation loading method employed a linear displacement loading approach consistent with the experimental loading procedure. A downward-enforced displacement of 1.5 mm was applied in the *y*-direction at the beam mid-span. An eight-node reduced integration element (C3D8R) was the element type employed for the simulation. Crack initiation was determined using the maximum principal stress criterion, and the initial crack interface was modelled as hard contact without friction. The initial crack configuration matched the pre-existing crack in the experiment, and a uniform global mesh size of approximately 0.005 m was applied. For models encountering convergence difficulties, adjustments such as modifying the damage stabilisation coefficient were implemented to improve convergence. The ABAQUS numerical model is shown in Figure 14.

### 6.2. Comparative Analysis of Simulation and Test Results

#### 6.2.1. The Cracking Process

**Figure 15:**
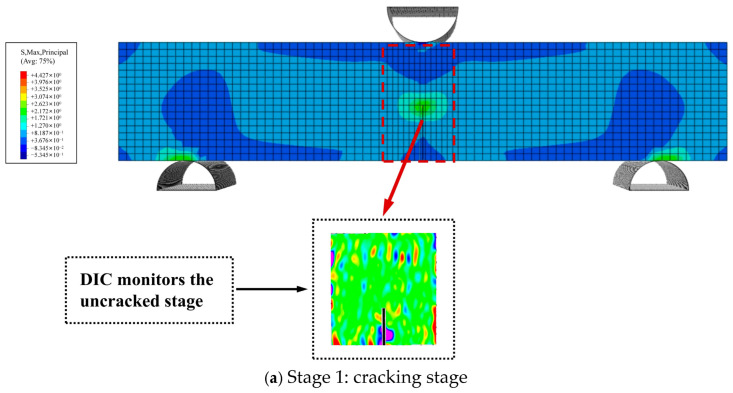

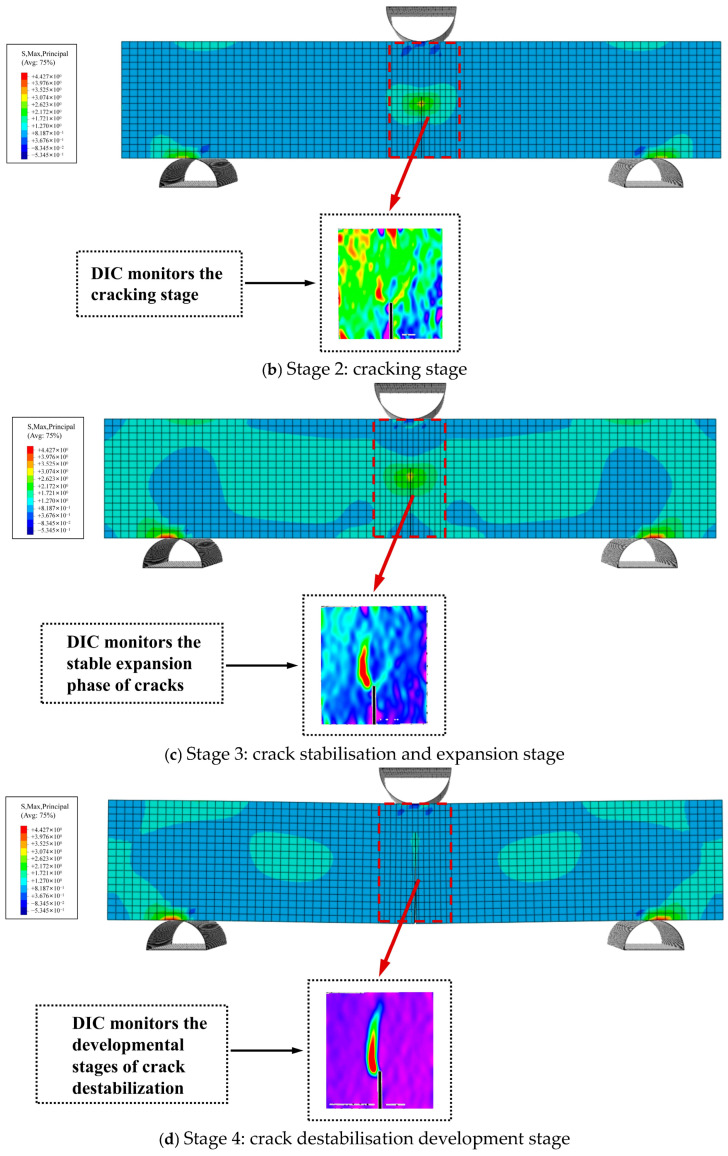
Maximum principal stress cloud for the cracking process of a three-point bending beam.

Figure 15 depicts the maximum principal-stress distribution during crack propagation in a three-point bending beam with an initial crack height ratio of 0.4. As the model was assumed to be a homogeneous solid, the crack propagation followed a straight path without deflection, aligning with the typical characteristics of a Mode I crack. During the early phase of crack propagation, the upper section of the beam primarily underwent compressive stress, whereas the region around the prefabricated crack at the bottom experienced tensile stress, resulting in stress concentration at the crack tip. As the applied displacement load increased, the tensile stress at the crack tip reached the maximum tensile strength of the material, thereby triggering crack initiation. The crack opening displacement increased accordingly, and the crack propagated vertically upward from the bottom centre. At the peak load, the region of maximum stress surrounding the crack path reached its largest possible extent, indicating that the model was reaching failure. Once the maximum principal stress reached its peak value, this high-stress region started to shrink, indicating that the model had lost its functionality and entered the unloading phase.

#### 6.2.2. Comparison of Force Curves

**Figure 16:**
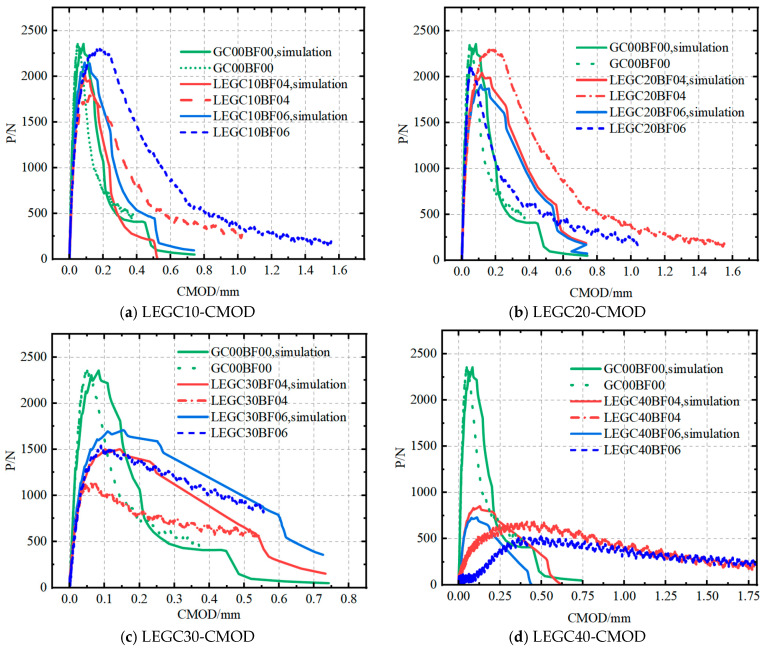
P-CMOD curves of concretes.

A comparison between the model predictions for various mix proportions and the experimental data yielded reaction force and crack-opening displacement curves, as illustrated in Figure 16.

As shown in Figure 16, the experimental and simulated P-CMOD curves exhibit nearly identical trends. However, for the same mix proportion, the peak loads obtained from the experiments for LEGC10, LEGC20, and LEGC30 were marginally higher than those obtained from the simulations. The primary reason for this discrepancy is that the simulation assumed the concrete beam to be an isotropic homogeneous solid, whereas in reality, the interlocking effect between aggregates enhances the material’s resistance to failure. Moreover, the crack-arresting and bridging effects of basalt fibres in the actual specimens contributed to the enhanced fracture performance of the concrete. Additionally, the incorporation of EPS particles modified the failure path, resulting in slightly higher experimental values than the predictions. However, for LEGC40, the experimental peak load was lower than the simulated value due to excessive EPS content. In the actual specimen, a higher number of EPS particles resulted in their detachment immediately after crack initiation, significantly reducing the fracture performance and reducing the experimental peak load below the average simulated value.

As shown in Table 5, the experimental and simulated peak loads of GC00BF00 exhibited a minor error of 0.11%. This minimal discrepancy was attributed to the concrete specimen with the mix proportion closely resembling an isotropic homogeneous solid. This further validates the accuracy of the model. The relatively large modelling error observed in LEGC40 can be attributed to the low density of EPS particles, which tend to float during the casting process, leading to non-uniform distribution within the specimen. This uneven dispersion is particularly pronounced at higher EPS contents, resulting in significant discrepancies between the experimental and simulated values. Similarly, the regression model predictions for LEGC10 and LEGC20 also showed small errors compared with the numerical simulation results. For instance, the errors for LEGC10BF06 and LEGC20BF04 were 0.92% and 0.97%, respectively, confirming the reliability of the linear regression relationship between concrete compressive strength and peak load. This indicates that the peak and crack initiation loads can be reliably estimated using the concrete compressive strength. Accordingly, a regression model was employed to predict the crack initiation load. Subsequently, the predicted values were incorporated into the numerical simulation. A comparison between the predicted crack initiation loads derived from the concrete strength and the experimental results further validated the reliability of the model. For GC00, LEGC10, and LEGC20, the errors in the crack initiation load were minimal, with LEGC10BF06 and LEGC20BF04 showing deviations of only 0.1% and 2.9%, respectively. Therefore, this model was considered reliable.

## 7. Fracture Key Parameter Prediction Equation

Research indicates that the fracture toughness is affected by variables such as concrete boundary conditions, size effects, and maximum aggregate particle size and distribution. When the geometric dimensions of the material remain constant, improvements in fracture toughness are closely related to strength. A stepwise regression analysis was performed by examining data from both the literature and this study to investigate the relationship among compressive strength, splitting tensile strength, and fracture toughness. The regression model reveals a strong linear correlation between the initial fracture toughness and compressive strength. However, for unstable fracture toughness, the correlation coefficient of the model was adjusted to R^2^ = 0.405, indicating that the independent variables explained only 40.5% of the variation in the unstable fracture toughness, which was below the 50.0% threshold, rendering the regression results unreliable.

**Figure 17:**
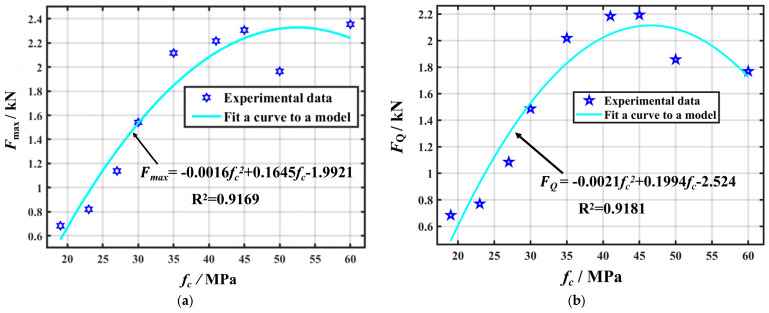
Regression curves on peak load and crack initiation load fitting. (**a**) Fitting relationship between peak load and compressive strength of concrete; (**b**) fitting relationship between concrete cracking load and compressive strength.

The initial cracking load can effectively characterise the fracture toughness, and its relationship with the compressive strength is particularly strong. Predicting the initial cracking load based on the compressive strength allows for a more accurate assessment of the concrete fracture toughness. Additionally, because measuring the initial cracking load is challenging, its prediction can enhance the accuracy of fracture toughness calculations. Similarly, unstable fracture toughness can be represented using the peak load, given its strong correlation with compressive strength. Through the analysis of experimental data, the compressive strength of the specimens was correlated with both the initial cracking load and peak load, and the corresponding fitted curves are illustrated in Figure 17.

As illustrated in Figure 11, the resulting regression model is as follows:

*F_Q_* = −0.0021*f_c_*^2^ + 0.1994*f_c_* − 2.524

*F_max_* = −0.0016*f_c_*^2^ + 0.1645*f_c_* − 1.9921

The correlation coefficients for the fitted curves were R^2^ = 0.9181 and 0.9169, indicating that the compressive strength explains 91.81% and 91.69% of the variations in the initial cracking load and peak load, respectively. This strong correlation indicates that the initial cracking load and peak load can be reliably estimated based on the compressive strength, enabling more accurate predictions of fracture toughness. However, the unstable fracture toughness is affected by both the load-bearing capacity and deformation ability of the material. Given that FPZ exhibited nonlinear deformation and cohesion distributions, the splitting tensile strength alone cannot explain the unstable fracture toughness. Nonetheless, predicting the peak load allows for a partial estimation of unstable fracture toughness. Moreover, accurate peak load predictions helped validate the experimental data against numerical simulations, thereby enabling a more comprehensive analysis of other fracture parameters and providing deeper insights into concrete fracture behaviour. By predicting the initial cracking load and peak load of concrete, the corresponding values of its unstable fracture toughness $K_{\mathrm{IC}}^{S}$ and initiation fracture toughness $K_{\mathrm{IC}}^{Q}$ can be estimated. The final predictive equations are derived as follows:

$$K_{\mathrm{IC}}^{S}\;=\;\frac{1.5(-0.0016{f_{c}}^{2}\;+\;0.1645f_{c}\;-\;1.9921\;+\;\frac{\mathrm{mg}}{2}\times 10^{-2})\times 10^{-3}Sa_{c}^{1/2}}{th^{2}}f(\alpha )$$

$$K_{\mathrm{IC}}^{Q}=\frac{1.5\left(-0.0021{f_{c}}^{2}\;+0.1994f_{c}\;-\;2.524+\frac{\mathrm{mg}}{2}\times 10^{-2}\right)\times 10^{-3}Sa_{0}^{\frac{1}{2}}}{th^{2}}f\left(\alpha^{‘}\right)$$

The calculated crack initiation load can be used to determine the crack initiation toughness after the numerical simulation. A comparison of the simulated and experimentally measured crack initiation toughness indicates that at a lower EPS particle content, the discrepancy between the two was minimal. However, as the EPS particle content increased, the error became more significant, particularly when the EPS volume fraction reached 40%. The primary reason for this is that excessive EPS content leads to poor dispersion, resulting in a less dense internal structure of the concrete. This structural deficiency affects the crack initiation load, which ultimately leads to a larger error in crack initiation toughness.

The analysis results indicate that the developed numerical model accurately replicates the mechanical response of the actual material. Moreover, the minimal deviation between the regression-based predictions and the simulation outcomes—both derived from compressive strength—verifies the robust linear relationship between compressive strength and peak load. This correlation offers a theoretical foundation for predicting the initial cracking load based on strength parameters in practical engineering applications. By integrating experimental observations, regression modelling, and numerical simulations, the study constructs a closed-loop predictive framework linking material characteristics to structural performance. This approach provides a quantitative basis for optimising mix design and defining the critical EPS content threshold in geopolymer lightweight concrete.

## 8. Conclusions

This study focused on evaluating the fracture performance of basalt fibre-reinforced LEGC with varying EPS volume fractions. Three-point bending tests were conducted on notched beam LEGC specimens, followed by calculations of initiation toughness, unstable toughness, fracture energy, and ductility index using DIC, X-CT scanning, and SEM analysis. The influences of basalt fibres and EPS particles on the fracture behaviour of this concrete were systematically investigated. Based on the experimental findings, the following conclusions were drawn:

1. The inclusion of EPS particles and basalt fibres had a substantial impact on the strength and fracture properties of geopolymer concrete. The fibres contributed to significant improvements in the initiation toughness, unstable fracture toughness, fracture energy, and ductility index of the EPS–geopolymer lightweight concrete. Their incorporation, along with the EPS particles, enhanced both the fracture energy and ductility index. This, in turn, improved the fracture resistance of the concrete. LEGC20BF04 exhibited the best fracture performance among all the specimens. Compared with GC00BF00, its initiation toughness, unstable toughness, fracture energy, and ductility index increased by 12.07%, 28.73%, 98.92%, and 111.27%, respectively.
2. The improvement in the fracture performance of the EPS–geopolymer lightweight concrete owing to the addition of basalt fibres was attributed to their influence on both the load-bearing capacity and deformation ability of the specimens. Basalt fibres compensated for the reduction in the initial cracking load caused by the EPS particles, which tended to weaken the concrete strength. By improving the ductility and deformation capacity of the geopolymer concrete during crack propagation, the basalt fibres enhanced the initiation toughness, unstable fracture toughness, fracture energy, and ductility. This delayed crack propagation instability, enabling the specimen to maintain a more stable fracture failure state.
3. By combining DIC, SEM, and micro-CT as multiscale characterisation techniques, the crack propagation mechanism and the reinforcing effect of basalt fibres were comprehensively revealed. DIC enabled full-field strain monitoring throughout the entire fracture process, from crack initiation to instability. SEM observations provided critical insights into fibre–matrix interfacial bonding and microcrack deflection behaviour, which directly correlate with the improved fracture toughness and energy absorption capacity observed in mechanical tests. Micro-CT facilitated three-dimensional visualisation of internal pore structures, crack distribution, and propagation paths. These analyses, from a microscale perspective, confirmed the crack-bridging and energy dissipation mechanisms of basalt fibres and their significant role in suppressing crack growth.
4. Crack development during the three-point bending of notched beams was numerically simulated using XFEM, accurately reproducing the crack propagation paths and fracture patterns under different mix proportions. A relational model was established linking the initial cracking load, peak load, and compressive strength, enabling rapid prediction of fracture toughness and fracture energy. This provides both theoretical support and a practical tool for the performance evaluation and structural design of this type of concrete.

This study investigated the static fracture performance of the proposed concrete, which demonstrates favourable fracture properties based on the experimental results. Future research may focus on exploring its dynamic fracture behaviour. Additionally, considering the excellent thermal insulation properties of EPS particles, it is recommended to further examine the mechanical performance of this concrete under freeze–thaw cycles.

## Figures and Tables

**Table 1:** Parameters of sodium silicate solution.

Na_2_O (%)	SiO_2_ (%)	H_2_O (%)	Ms (SiO_2_/Na_2_O)	Pomerol (Be/68 °F)	Specific Gravity
8.54	27.30	64.16	3.30	38.5	1.37

**Table 2:** Mix proportions of specimens.

Specimen Identification	Mixing Ratio of Main Ingredients (kg/m^3^)						Expanded Polystyrene (EPS)		Basalt Fibre (BF)		Polycarboxylic Acid High-Efficiency Water-Reducing Agent (kg/m^3^)
	Slag	FA	NaOH	Na_2_SiO_3_	Sand	H_2_O	Weight (kg/m^3^)	Volume (%)	Weight (kg/m^3^)	Volume (%)	
GC00BF00	348.8	348.8	45.2	216.9	1011.8	118.0	-	-	-	0	10.5
LEGC10BF04	348.8	348.8	45.2	216.9	811.8	118.0	1.8	10	10.6	0.4	10.5
LEGC10BF06	348.8	348.8	45.2	216.9	811.8	118.0	1.8	10	15.9	0.6	10.5
LEGC20BF04	348.8	348.8	45.2	216.9	611.8	118.0	3.7	20	10.6	0.4	10.5
LEGC20BF06	348.8	348.8	45.2	216.9	611.8	118.0	3.7	20	15.9	0.6	10.5
LEGC30BF04	348.8	348.8	45.2	216.9	411.8	118.0	5.5	30	10.6	0.4	10.5
LEGC30BF06	348.8	348.8	45.2	216.9	411.8	118.0	5.5	30	15.9	0.6	10.5
LEGC40BF04	348.8	348.8	45.2	216.9	211.8	118.0	7.3	40	10.6	0.4	10.5
LEGC40BF06	348.8	348.8	45.2	216.9	211.8	118.0	7.3	40	15.9	0.6	10.5

**Table 3:** Experimental parameter analysis table.

Specimen Number	*F_max_*/kN	*F_Q_*/kN	*CMOD*/mm	*δ_max_*/mm	*P-δ*/J
GC00BF00	2.355	1.769	0.05	1.649	821.71
LEGC10BF04	1.965	1.858	0.144	2.477	970.80
LEGC10BF06	2.307	2.195	0.179	2.512	1783.39
LEGC20BF04	2.217	2.185	0.185	2.484	1783.56
LEGC20BF06	2.116	2.019	0.196	2.772	1606.88
LEGC30BF04	1.278	1.085	0.176	2.151	1163.33
LEGC30BF06	1.541	1.487	0.190	3.470	2077.79
LEGC40BF04	0.684	0.622	0.350	3.590	1289.70
LEGC40BF06	0.82	0.77	0.391	2.812	930.77

where *F_max_* is peak load; *F_Q_* is incidence of cracking load; *CMOD* is crack end tension displacement; *δ_max_* is maximum displacement; *P-δ* is area enclosed by load and displacement curves and horizontal coordinates.

**Table 4:** Model parameter values.

Specimen Number	Density (kg/m^3^)	Modulus of Elasticity (GPa)	Poisson’s Ratio	Rupture Energy (N/m)
GC00BF00	2114.4	7.6	0.11	166.98
LEGC10BF04	1912.6	6.2	0.16	212.39
LEGC10BF06	1980.6	6.2	0.16	339.08
LEGC20BF04	1669.9	5.5	0.18	332.15
LEGC20BF06	1747.6	5.5	0.18	309.24
LEGC30BF04	1475.7	4.8	0.21	298.49
LEGC30BF06	1485.4	4.8	0.21	389.37
LEGC40BF04	1048.5	3.9	0.25	246.61
LEGC40BF06	1097.6	3.9	0.25	147.73

**Table 5:** Experimental and simulation results.

Specimen Number	*F_max_*/kN			*F_Q_*/kN		$K_{IC}^{Q}$ $/\mathrm{MPa}.\sqrt{m}$		Inaccuracies (%)
	Experimental Values	Model Prediction	Numerical Simulations	Experimental Values	Model Prediction	Experimental Values	Numerical Simulations	
GC00BF00	2.355	2.118	2.353	1.769	1.88	0.58	0.608	4.6
LEGC10BF04	1.965	2.232	2.011	1.858	2.196	0.54	0.67	19.4
LEGC10BF06	2.307	2.170	2.15	2.195	2.197	0.66	0.676	2.4
LEGC20BF04	2.217	2.063	2.043	2.185	2.121	0.65	0.637	2.0
LEGC20BF06	2.116	1.805	1.90	2.019	1.882	0.64	0.584	8.7
LEGC30BF04	1.278	1.283	1.502	1.085	1.328	0.37	0.430	13.9
LEGC30BF06	1.541	1.503	1.707	1.487	1.568	0.47	0.486	3.3
LEGC40BF04	0.684	0.556	0.851	0.622	0.507	0.24	0.194	19.2
LEGC40BF06	0.82	0.945	0.98	0.77	0.951	0.26	0.307	15.3

where *F_max_* is peak load; *F_Q_* is incidence of cracking load; $K_{IC}^{Q}$ is cracking toughness.

## Data Availability

The original contributions presented in this study are included in the article. Further inquiries can be directed to the corresponding author.

## References

[1] Tang W.C., Balendran R.V., Nadeem A., Leung H.Y. (2006). Flexural strengthening of reinforced lightweight polystyrene aggregate concrete beams with near-surface mounted GFRP bars. Build. Environ..

[2] Liu H., Han J., Parsons R.L. (2021). Mitigation of seasonal temperature change-induced problems with integral bridge abutments using EPS foam and geogrid. Geotext. Geomembr..

[3] Sulong N.H.R., Mustapa S.A.S., Rashid M.K.A. (2019). Application of expanded polystyrene (EPS) in buildings and constructions: A review. J. Appl. Polym. Sci..

[4] Moradikhou A.B., Esparham A., Avanaki M.J. (2020). Physical & mechanical properties of fiber reinforced metakaolin-based geopolymer concrete. Constr. Build. Mater..

[5] Kim G.W., Oh T., Lee S.K., Banthia N., Yoo D.-Y. (2023). Development of Ca-rich slag-based ultra-high-performance fiber-reinforced geopolymer concrete (UHP-FRGC): Effect of sand-to-binder ratio. Constr. Build. Mater..

[6] Provis J.L., Yong C.Z., Duxson P., van Deventer J.S.J. (2009). Correlating mechanical and thermal properties of sodium silicate-fly ash geopolymers. Colloids Surf. A Physicochem. Eng. Asp..

[7] Kanagaraj B., Anand N., Samuvel R.R., Lubloy E. (2022). Performance evaluation of sodium silicate waste as a replacement for conventional sand in geopolymer concrete. J. Clean. Prod..

[8] Aguilar R.A., Díaz O.B., García J.I.E. (2010). Lightweight concretes of activated metakaolin-fly ash binders, with blast furnace slag aggregates. Constr. Build. Mater..

[9] Sanjayan J.G., Nazari A., Chen L., Nguyen G.H. (2015). Physical and mechanical properties of lightweight aerated geopolymer. Constr. Build. Mater..

[10] Bhutta M.A.R., Ohama Y., Tsuruta K. (2011). Strength Properties of Polymer Mortar Panels Using Methyl Methacrylate Solution of Waste Expanded Polystyrene as Binder. Constr. Build. Mater..

[11] Chen B., Liu J. (2007). Mechanical properties of polymer-modified concretes containing expanded polystyrene beads. Constr. Build. Mater..

[12] Shirai K., Horii J., Nakamuta K., Teo W. (2022). Experimental investigation on the mechanical and interfacial properties of fiber-reinforced geopolymer layer on the tension zone of normal concrete. Constr. Build. Mater..

[13] Bhutta A., Borges P.H.R., Zanotti C., Farooq M., Banthia N. (2017). Flexural behavior of geopolymer composites reinforced with steel and polypropylene macro fibers. Cem. Concr. Compos..

[14] Yu W., Jin L., Du X. (2023). Experimental study on compression failure characteristics of basalt fiber-reinforced lightweight aggregate concrete: Influences of strain rate and structural size. Cem. Concr. Compos..

[15] Xie H., Yang L., Zhang Q., Huang C., Chen M., Zhao K. (2022). Research on energy dissipation and damage evolution of dynamic splitting failure of basalt fiber reinforced concrete. Constr. Build. Mater..

[16] Zhang H., Wang B., Xie A., Qi Y. (2017). Experimental study on dynamic mechanical properties and constitutive model of basalt fiber reinforced concrete. Constr. Build. Mater..

[17] Saloni, Parveen, Pham T.M. (2020). Enhanced properties of high-silica rice husk ash-based geopolymer paste by incorporating basalt fibers. Constr. Build. Mater..

[18] Şahin F., Uysal M., Canpolat O., Aygörmez Y., Cosgun T., Dehghanpour H. (2021). Effect of basalt fiber on metakaolin-based geopolymer mortars containing rilem, basalt and recycled waste concrete aggregates. Constr. Build. Mater..

[19] Bencardino F., Rizzuti L., Spadea G., Swamy R.N. (2013). Implications of test methodology on post-cracking and fracture behaviour of steel fibre reinforced concrete. Compos. Part B Eng..

[20] Yin S., Tuladhar R., Collister T., Combe M., Sivakugan N., Deng Z. (2015). Post-cracking performance of recycled polypropylene fibre in concrete. Constr. Build. Mater..

[21] Ferdosian I., Camões A. (2021). Mechanical performance and post-cracking behavior of self-compacting steel-fiber reinforced eco-efficient ultra-high performance concrete. Cem. Concr. Compos..

[22] Yoo D.Y., Yoon Y.S., Banthia N. (2015). Predicting the post-cracking behavior of normal-and high-strength steel-fiber-reinforced concrete beams. Constr. Build. Mater..

[23] Fan B., Qiao Y., Hu S. (2020). An experimental investigation on FPZ evolution of concrete at different low temperatures by means of 3D-DIC. Theor. Appl. Fract. Mech..

[24] Guo Q., Wang H., Gao Y., Jiao Y., Liu F., Dong Z. (2020). Investigation of the low-temperature properties and cracking resistance of fiber-reinforced asphalt concrete using the DIC technique. Eng. Fract. Mech..

[25] Li Q.H., Yin X., Huang B.T., Luo A.M., Lyu Y., Sun C.J., Xu S.L. (2021). Shear interfacial fracture of strain-hardening fiber-reinforced cementitious composites and concrete: A novel approach. Eng. Fract. Mech..

[26] Bhosale A.B., Lakavath C., Prakash S.S. (2020). Multi-linear tensile stress-crack width relationships for hybrid fibre reinforced concrete using inverse analysis and digital image correlation. Eng. Struct..

[27] Wei J., Yang Q.S. (2023). Mechanical Properties of Basalt Fiber-Reinforced Ambient-Cured Novel Lightweight Eps Geopolymer Concrete. J. Build. Eng..

[28] (2008). Granulated Blast Furnace Slag Used for Cement Production.

[29] (1997). Chemical Reagent. Sodium Hydroxide.

[30] (2017). Standard Test Method for Relative Density (Specific Gravity) and Absorption of Fine Aggregate.

[31] Skarżyński Ł., Tejchman J. (2013). Experimental investigations of fracture process using DIC in plain and reinforced concrete beams under bending. Strain.

[32] Suchorzewski J., Tejchman J., Nitka M. (2018). Experimental and numerical investigations of concrete behaviour at meso-level during quasi-static splitting tension. Theor. Appl. Fract. Mech..

[33] Suchorzewski J., Tejchman J., Nitka M. (2018). Discrete element method simulations of fracture in concrete under uniaxial compression based on its real internal structure. Int. J. Damage Mech..

[34] Skarżyński Ł., Marzec I., Tejchman J. (2019). Fracture evolution in concrete compressive fatigue experiments based on X-ray micro-CT images. Int. J. Fatigue.

[35] Skarżyński Ł., Tejchman J. (2016). Experimental investigations of fracture process in concrete by means of X-ray micro-computed tomography. Strain.

[36] Skarżyński Ł., Suchorzewski J. (2018). Mechanical and fracture properties of concrete reinforced with recycled and industrial steel fibers using Digital Image Correlation technique and X-ray micro computed tomography. Constr. Build. Mater..

[37] Skarżyński Ł. (2020). Mechanical and radiation shielding properties of concrete reinforced with boron-basalt fibers using Digital Image Correlation and X-ray micro-computed tomography. Constr. Build. Mater..

[38] Ponikiewski T., Katzer J., Bugdol M., Rudzki M. (2015). X-ray computed tomography harnessed to determine 3D spacing of steel fibres in self compacting concrete (SCC) slabs. Constr. Build. Mater..

[39] Desmorat R., Gatuingt F., Ragueneau F. (2007). Nonlocal anisotropic damage model and related computational aspects for quasi-brittle materials. Eng. Fract. Mech..

[40] Grassl P., Xenos D., Nyström U., Rempling R., Gylltoft K. (2013). CDPM2: A damage-plasticity approach to modelling the failure of concrete. Int. J. Solids Struct..

[41] Ortiz M., Pandolfi A. (1999). Finite-deformation irreversible cohesive elements for three-dimensional crack-propagation analysis. Int. J. Numer. Methods Eng..

[42] Zhou F., Molinari J.F. (2004). Dynamic crack propagation with cohesive elements: A methodology to address mesh dependency. Int. J. Numer. Methods Eng..

[43] Xue C., Li W., Li J., Wang K. (2019). Numerical investigation on interface crack initiation and propagation behaviour of self-healing cementitious materials. Cem. Concr. Res..

[44] Wells G.N., Sluys L.J. (2001). A new method for modelling cohesive cracks using finite elements. Int. J. Numer. Methods Eng..

[45] Moës N., Belytschko T. (2002). Extended finite element method for cohesive crack growth. Eng. Fract. Mech..

[46] Siddique R., Mehta A. (2014). Effect of carbon nanotubes on properties of cement mortars. Constr. Build. Mater..

[47] Xu S.L., Reinhardt H.W. (1999). Determination of double-K criterion for crack propagation in quasi-brittle fracture, part ll:Analytical evaluating and practical measuring methods for three-point bending notched beams. J. Int. J. Fracture..

[48] (1985). Determination of the fracture energy of mortar and concrete by means of three-point bend tests on notched beams. Mater. Structures.

